# Supplementary material for: Structure/Function Studies on the Activation Motif of Two Non-Mammalian Mrap1 Orthologs, and Observations on the Phylogeny of Mrap1, Including a Novel Characterization of an Mrap1 from the Chondrostean Fish, Polyodon spathula
Source: Biomolecules. 2022 Nov 12;12(11):1681. doi: 10.3390/biom12111681 (PMC9688151; doi:10.3390/biom12111681)
Supplement: Supplementary file 1 [file biomolecules-12-01681-s001.zip › biomolecules-2015407-supplementary.pdf]

Figure S1A: Alanine Mutants of cMRAP1

cMRAP1	MANRTNSSEYFWSYEYYWDYIDPIPVDGRKLKVNKY
A18A19A20A21	MANRTNSSEYFWSYEYYAAAADPIPVDGRKLKVNKY
W18/A18	MANRTNSSEYFWSYEYYADYIDPIPVDGRKLKVNKY
D19/A19	MANRTNSSEYFWSYEYYWAYIDPIPVDGRKLKVNKY
Y20/A20	MANRTNSSEYFWSYEYYWDAIDPIPVDGRKLKVNKY
I21/A21	MANRTNSSEYFWSYEYYWDYADPIPVDGRKLKVNKY

Figure S1B: Alanine Mutants of bfMrap1

bfMrap1	MKNNTNTSEYVWTYEYFYDYIDPVVVDEKQLRFNKY
A18A19A20A21	MKNNTNTSEYVWTYEYFAAAADPVVVDEKQLRFNKY
Y18/A18	MKNNTNTSEYVWTYEYFADYIDPVVVDEKQLRFNKY
D19/A19	MKNNTNTSEYVWTYEYFYAYIDPVVVDEKQLRFNKY
Y20/A20	MKNNTNTSEYVWTYEYFYDAIDPVVVDEKQLRFNKY
I21/A21	MKNNTNTSEYVWTYEYFYDYADPVVVDEKQLRFNKY
